# RACIAL AND RURAL-URBAN DISPARITIES IN DISCHARGE AMONG MEDICARE PATIENTS HOSPITALIZED FOR ISCHEMIC STROKE

**DOI:** 10.1093/geroni/igae098.4060

**Published:** 2024-12-31

**Authors:** Mengyuan Cheng, Winston Kennedy, Nasim Ferdows

**Affiliations:** Northeastern University, Boston, Massachusetts, United States; Northeastern University, Boston, Massachusetts, United States; Northeastern University, Boston, Massachusetts, United States

## Abstract

Disparities in post-acute stroke care are a critical public health issue, as they can profoundly affect patient outcomes and long-term recovery. Understanding how race and rurality shape discharge destination patterns is essential for addressing these inequities. This study explores these disparities among Medicare beneficiaries hospitalized for ischemic stroke in 2021, using data from the 5% Medicare Fee-For-Service sample. We conducted multinomial logistic regression, stratifying by survival status and adjusting for age, sex, stroke severity, comorbidity, dual-eligibility status, depression, hospital length of stay, and state Medicaid expansion status. Rurality was categorized using Rural-Urban Commuting Area codes into urban, rural-adjacent, and rural non-adjacent areas. Significant disparities emerged among patients who died during or shortly after hospitalization. Hispanic and Black patients had 62% lower odds of being discharged to hospice, another hospital, or other settings compared to White patients (OR=0.38, 95% CI 0.20-0.73). Patients classified as ‘Others’ (Asian, Native American, etc.) had 74% lower odds of being discharged to such settings (OR=0.26, 95% CI 0.11-0.62). Rural-adjacent patients had 44% lower odds of being discharged to hospice or other settings compared to urban patients (OR=0.56, 95% CI 0.31-1.01), while rural non-adjacent patients showed a 36% reduction in odds, though this was not statistically significant. Our findings highlight the critical need for tailored interventions to address racial and rural-urban disparities in post-stroke care, particularly for patients who die during or shortly after hospitalization, to promote equitable care.

